# Prevalence, Severity, and Treatment of Obstructive Sleep Apnea in Children with Down Syndrome in Qatar

**DOI:** 10.7759/cureus.61777

**Published:** 2024-06-06

**Authors:** Amal R Al-Naimi, Mutasim Abu-Hasan, Antonisamy Belavendra, Ibrahim Janahi

**Affiliations:** 1 Pediatric Pulmonology, Sidra Medicine, Doha, QAT; 2 Pediatrics, Sidra Medicine, Doha, QAT

**Keywords:** central apnea, polysomnography, obstructive sleep apnea, sleep disorder breathing, down syndrome

## Abstract

Introduction: Patients with Down syndrome (DS) are at risk for sleep disorder breathing (SDB) due to their abnormal craniofacial anatomy, hypotonia, and propensity for obesity. The prevalence and severity of SDB in this population vary between different cohorts due to the multifactorial nature of these patients and the different diagnostic criteria used. We aim to report the prevalence and severity of SDB in the DS population in Qatar.

Methods: This study is a retrospective review of all patients with genetically confirmed DS who completed a diagnostic polysomnography (PSG) study at Sidra Medicine in Doha, Qatar, which is the only pediatric sleep center in the country, between September 2019 and July 2022. Clinical and PSG data were collected from the patients’ electronic medical records. Central and obstructive events were scored according to the American Academy of Sleep Medicine (AASM) criteria. Obstructive sleep apnea (OSA) diagnosis was made based on apnea-hypopnea index (AHI) and defined as AHI >1.5 events/hour. OSA was considered mild if AHI was ≥ 1.5 but < 5, moderate if AHI was ≥ 5 but < 10, and severe if AHI was ≥ 10 events/hour. Diagnosis with central apnea was considered if the central apnea index was > 5 events/hour. Hypoventilation was considered present if end-tidal/transcutaneous carbon dioxide gas was more than 50 mmHg for more than 25% of total sleep time. Multiple regression analysis was performed to evaluate predictors of high AHI and rapid eye movement (REM)-AHI.

Results: A total of 80 patients (49 males and 31 females) were included. Median (range) age was 7.3 years (0.9, 21). The mean (range) BMI z-score was 1.7 (-1.3, 4.3). Sixty-five patients were diagnosed with OSA, with a prevalence rate of 81%. OSA was mild in 25 (38.5%) patients, moderate in 15 (23.1%) patients, and severe in 25 (38.5%) patients. Only one patient was diagnosed with central apnea and five patients (6.9%) with alveolar hypoventilation. Multiple regression analysis showed BMI (P = 0.007) and snoring/apnea symptoms (P=0.023) to be predictive of high AHI. No correlation was found between the same variables and REM-AHI. Treatments used for OSA included anti-inflammatory medications in 37 (46%) patients, tonsillectomy/adenoidectomy in 13 (16.5%) patients, and positive airway pressure support in 10 (15%) patients.

Conclusion: Our patient population with DS had a high prevalence of OSA comparable to other reported cohorts. High BMI and symptoms of snoring are predictive of OSA.

## Introduction

Down syndrome (DS) is the most common chromosome abnormality among newborns with an estimated incidence of 20 cases per 10,000 live births [1]. Patients with DS have significant respiratory, cardiac, gastrointestinal, neurological, musculoskeletal, immune, and endocrine comorbidities. Sleep-disordered breathing (SDB), especially obstructive sleep apnea (OSA), is a very common and potentially serious respiratory problem in DS. The presence of hypotonia, midface hypoplasia, macroglossia, tonsillar and adenoidal hypertrophy, and truncal obesity contribute to their increased prevalence and severity [2].

Untreated OSA is shown to be associated with long-term behavioral and learning problems, impaired growth, and cardiovascular complications [3,4]. The American Academy of Pediatrics (AAP) recommends routine evaluation of all children with DS for OSA using polysomnography (PSG) starting at four years of age, regardless of the presence of symptoms [5]. Variations among the different risk factors for OSA in patients with DS can potentially result in differences in the prevalence and severity of OSA among different cohorts. Also, a lack of agreement on the diagnostic criteria for each type of SDB can also result in that discrepancy. The study of different patient cohorts is important and can help us better understand the specific contributing factors to SDB in each cohort and to individualize the diagnostic and therapeutic approach.

Therefore, our objective in this study was to evaluate the prevalence and the severity of the different types of SDB including OSA, central sleep apnea (CSA), and central hypoventilation in children with DS in the state of Qatar, which has not been previously reported, to our knowledge. We also aim to investigate the clinical and demographic factors that correlate with the disease severity and investigate the type of treatments needed for these disorders.

## Materials and methods

We retrospectively reviewed all children with a genetically confirmed diagnosis of DS who completed at least one diagnostic polysomnography (PSG) in the Pediatric Sleep Laboratory at Sidra Medicine Hospital, Doha, Qatar, between September 1, 2019, and July 30, 2022. Our lab is the only pediatric sleep lab in the country. Patients with poor sleep efficiency of ≤ 40% and patients with incomplete PSG respiratory signal data were excluded.

The following demographic and clinical data were collected from electronic medical records: age, gender, BMI, BMI z score, symptoms of snoring and/or witnessed sleep apnea, other clinical comorbidities (i.e., congenital heart disease (CHD), gastroesophageal reflux, asthma, allergic rhinitis, pulmonary hypertension), reported adenoid/tonsillar hypertrophy, history of adenotonsillectomy (A&T), persistent OSA post A&T. Need for nighttime respiratory support including continuous positive airway pressure (CPAP), bilevel positive airway pressure (BiPAP), and nasal O2 post PSG were also collected.

The following PSG parameters were collected: sleep efficiency, AHI, REM-AHI, mean O2 saturation, nadir O2 saturation, end-tidal CO2, and/or transcutaneous CO2, if available. All obstructive and central apnea and hypopnea events were scored according to the American Academy of Sleep Medicine (AASM) criteria by a trained lab technician. Diagnosis with OSA was made if AHI ≥ 1.5 events/hour. OSA was considered mild if AHI was > 1.5 but < 5 events/hour, moderate if AHI was > 5 but < 10 events/hour, and severe if AHI was ≥ 10 events/hour. CSA was considered present if the patient had central apnea events more frequently than five events/hour. Diagnosis of hypoventilation was considered if transcutaneous CO2 was > 50 mmHg for more than 25% of the total sleep time [6].

Demographic and clinical characteristics were summarized as mean and standard deviation (SD) for normally distributed continuous variables and median (range) for skewed continuous variables. Scatterplots of age and BMI against AHI were constructed. Similar plots were constructed for age and BMI against REM-AHI. Spearman rank correlation was used to determine the significance of correlation between the skewed variables. Positively skewed variables (AHI and REM-AHI) were log-transformed. The relationships between logged outcome variables (AHI and REM-AHI) and age, gender, BMI, and symptomatic and fixed CHD were assessed using univariable and multivariate linear regressions with all variables included simultaneously. Statistical analysis was performed using STATA IC/16.0 (StataCorp LLC, College Station, Texas, United States).

## Results

A total of 80 patients (49 males and 31 females) with a confirmed diagnosis of DS/Trisomy 21 were identified from patient records and were included in the study. The median (range) age was 7.3 years (0.9, 21). The mean (range) BMI z-score was 1.7 (-1.3, 4.3). Twenty-nine (36.3%) patients had CHD which was surgically corrected in all patients before PSG was performed. Only one patient was diagnosed with pulmonary hypertension by cardiac catheterization and was receiving anti-pulmonary hypertensive medications when the sleep study was done. The majority of patients were reportedly symptomatic (63 patients, 78.8%); 35 patients had a history of snoring, one patient had a history of witnessed apnea, and 27 patients had a history of both snoring and witnessing sleep apnea. Detailed demographic and clinical data are shown in Table 1.

**Table 1:** Clinical characteristics of the study participants (N=80) Data given as median (range) and n (%)

Characteristics	Total
Age (years), median (range)	7.3 (0.9, 21)
Gender (M/F), n (%)	49/31 (61.2/38.8)
BMI (kg/m^2^), median (range)	17.7 (12.2, 49.6)
BMI z-score, median (range)	1.7 (-1.3, 4.3)
Symptomatic, n (%)	63/80 (78.8)
Only snoring, n (%)	35/63 (55.6)
Only witnessed sleep apnea, n (%)	1/63 (1.6)
Snoring and witnessed sleep apnea, n (%)	27/63 (42.9)
Comorbidities, n (%)	
Asthma	10 (12.5)
Allergic rhinitis	5 (6.3)
Gastroesophageal reflux disease	9 (11.3)
Congenital heart disease	29 (36.3)
Pulmonary hypertension	1 (1.3)
Adenotonsillectomy, n(%)	13 (16.5)

Median (range) sleep efficiency for all patients was 78.7% (41%, 97%). Median (range) sleep latency was 32.7 (0, 236.5) minutes. Median (range) REM latency was 128.5 (1, 385.5) minutes. Median (range) wakefulness after sleep onset was 52.3 (4.5, 256) minutes. Mean±SD percentage of REM during sleep was 22.5±8.5. Median (range) AHI was 5.0 (0, 125) events/hour. Median (range) REM-AHI was 10.8 (0, 95) events/hour. The overall mean oxygen saturation was 95% and the mean of nadir oxygen saturation was 85%. Snoring was reported during PSG in 70 patients (87%). Sixty-five patients were diagnosed with OSA with estimated prevalence rate of 81%. OSA was mild in 25 (38.5%) patients, moderate in 15 (23.1%) patients, and severe in 25 (38.5%) patients. Five patients (6.9%) were diagnosed with alveolar hypoventilation. Only one patient had high central apnea index > 5 central apnea events/hour) but was associated with severe OSA (AHI of 34 events/hour). PSG data are detailed in Table 2.

**Table 2:** Polysomnography data (N=80) Data given as median (range), mean±SD, and n (%) REM: rapid eye movement; WASO: wakefulness after sleep onset; AHI: apnea-hypopnea index; OSA: obstructive sleep apnea; EtCO₂: end-tidal carbon dioxide; SpO2: saturation of peripheral oxygen

Findings	Values
Sleep efficiency, median (range)	78.7 (41.1, 97.1)
Sleep latency, median (range)	32.7 (0, 236.5)
REM latency, median (range)	128.5 (1, 385.5)
WASO, median (range)	52.3 (4.5,256)
REM%, mean±SD	22.5±8.5)
AHI, median (range)	5.0 (0, 125)
Patients with OSA, n (%)	65/80 (81.3)
Mild OSA	25/65 (38.5)
Moderate OSA	15/65 (23.1)
Severe OSA	25/65 (38.5)
REM-AHI, median (range)	10.8 (0, 95)
Snoring events, n (%)	70 (87.5)
O2 sat, mean± SD	95.7±2.8)
O2 nadir, mean± SD	85.8±6.6)
Percentage of total sleep time with SpO2 <90%, median (range)	0.1 (0, 99.7)
Peak EtCO₂, mean± SD	49.2±5.3)
Hypoventilation, n (%)	5/72 (6.9)
Nocturnal respiratory support, n (%)	7/80 (8.7)

Single variable regression analysis showed significant positive correlation between AHI and each of the following variables: age (P = 0.001), BMI (P < 0.001), history of snoring/apnea symptoms (P = 0.011) and history of CHD (P = 0.025). However, multiple regression analysis showed BMI (P = 0.007) and snoring/apnea symptoms (P=0.023) as the only significant predictors of high AHI. None of the above-mentioned variables were significant predictors of REM-AHI (Table 3). The interaction between age and BMI on the outcome variables was examined and none were significant.

**Table 3:** Multiple regression analysis for AHI and REM-AHI Estimates of effect size are presented as odds ratios and 95% confidence intervals as exponentiated β-coefficients (e^β^) for the continuous logged variables for AHI and REM-AHI. Exponentiated β-values indicate the percentage change in the outcome per unit change in the predictor variable Models were adjusted for age, gender, BMI, symptomatic and fixed CHD. CHD: congenital heart disease; AHI: apnea-hypopnea index; REM: rapid eye movement

Variables	Logged AHI		Logged REM-AHI	
	e ^β ^(95%CI)	p-value	e ^β ^(95%CI)	p-value
Age, years	1.03 (0.9, 1.1)	0.409	1.04 (0.9, 1.1)	0.344
Gender (male)	1.06 (0.6, 1.8)	0.825	1.06 (0.6, 1.8)	0.828
BMI	1.06 (1.0, 1.1)	0.007	1.03 (0.9, 1.1)	0.141
Symptomatic (yes)	2.07 (1.1, 3.9)	0.023	1.28 (0.7, 2.4)	0.434
Fixed CHD (yes)	0.69 (0.4, 1.2)	0.181	0.80 (0.5, 1.4)	0.405

A total of 37 (46%) patients received medical treatment for OSA with oral montelukast and nasal steroids. A total of 13 (16.5%) patients had A&T prior to PSG, but 12 out of these 13 patients had persistent OSA: three (25%) patients had mild OSA, three (25%) had moderate OSA, and six (50%) had severe OSA.

A total of 10 (15%) patients needed some form of respiratory support to treat OSA: one patient required nighttime O2 by nasal canula only, seven patients required nighttime CPAP, and two patients required nighttime BiPAP. Two (20%) of the patients who required any respiratory support had moderate OSA and eight (32%) patients had severe OSA. Twenty-two (33.8%) patients of the total cohort did not require any intervention, 11 (44%) of these untreated patients had mild OSA, four (27%) had moderate OSA, and seven (28%) had severe OSA.

## Discussion

Our study showed a high prevalence of OSA in DS at an estimated rate of 81.3%, with moderate to severe disease in 61.5% of the patients, which is similar to the prevalence reported in other patient cohorts. In a meta-analysis of all published studies of OSA in DS, overall prevalence was estimated at a rate of 69% when OSA was defined as AHI > 1 events/hour, 76% when OSA was defined as AHI> 1.5 events/hour, and 75% when OSA was defined as AHI> 2 events/hour [7]. The study also estimated that 50% of the patients have moderate-to-severe OSA, which is similar to the rate we found in our patient population.

Using single variable regression analysis, we found that age, BMI, snoring/apnea symptoms, and history of CHD had a significantly positive correlation with AHI. However, using multiple regression analysis, only BMI and history of snoring/apnea were significant predictors of high AHI. Several studies were done to evaluate the various risk factors of OSA in patients with DS but showed inconsistent and sometimes contradictory results. A recent systemic review of all published studies evaluating predictors of high AHI in DS found a significant positive correlation with age in two out of nine studies, but a negative correlation with age in two other studies [8]. There was no significant correlation between AHI and age in five other studies. In our study, age was not a significant risk factor for AHI in the multivariable analysis. This discrepancy between the different studies could be explained by the difference in age distribution among their study patients. In DS, age-related factors may play different roles in OSA. For example, hypotonia and micrognathia play a more significant role in younger patients with DS, while obesity plays a more significant role in older children. Our study included only two patients below the age of two years. Most of our study patients were four years or older since our center follows the AAP recommendation for screening patients with DS with PSG starting at four years of age unless symptomatic [5]. Moreover, differences in the risk factors for OSA among the different cohorts of DS can also be related to differences in research methodologies and variations in OSA definition, as discussed above.

Our study showed that symptoms of snoring and apnea were strong predictors of high AHI in DS. In the previously mentioned systematic review, only two studies evaluated the predictive value of snoring history, and only one of them showed a significant correlation. On the other hand, it is important to note that the absence of any history of snoring or witnessed apneas does not necessarily rule out OSA in DS. Maris et al. reported an OSA prevalence of 53.8% in patients with DS who were older than four years of age and who did not have any clinical symptoms [2]. This further supports the AAP recommendations to routinely obtain sleep studies in all individuals with DS regardless of symptoms.

Different therapies have been used to treat OSA in our patient population including the use of anti-inflammatory mediators, adenotonsillectomy, and/or positive airway pressure support [9-11]. We found a trend towards the use of anti-inflammatories in mild OSA, adenotonsillectomy in moderate to severe OSA, and CPAP/BiPAP in severe OSA patients but not consistently. There was no clear treatment protocol that was strictly followed. For example, we had seven patients with severe OSA who did not receive any treatment. This variation in treatments highlights the importance of using an evidence-based approach to treat OSA in DS.

The effectiveness of different treatment modalities for OSA in DS remains to be adequately determined. A&T, which is the most effective therapy for OSA in the pediatric general population is not as effective in treating OSA in DS. Several studies reported persistent OSA in 30-50% of patients with DS post A&T, strongly suggesting that tonsillar and adenoid enlargement are not the only major predisposing factor for OSA in DS. Multiple studies using cine-MRI showed that macroglossia, glossoptosis, and lingual tonsil hypertrophy are possible causes of persistent OSA [12,13]. The results of these studies emphasized the need for close follow-up post adenotonsillectomy, and escalation of therapy in patients with persistent and severe symptoms post-surgery. Individualized diagnostic approaches for such patients to evaluate the etiology of the upper airway obstruction using drug-induced sleep endoscopy (DISE) and/or sleep/cine MRI have been recommended to identify other surgically treatable causes of upper airway obstruction [14,15]. Positive airway pressure (CPAP/BiPAP) should be considered in patients who fail surgical treatments.

Large studies evaluating short-term and long-term effects of positive airway therapy in patients with persistent OSA are badly needed. Our study was a retrospective review and included DS patients who were referred for OSA assessment. 

## Conclusions

OSA is a very common respiratory problem in patients with DS. Various interacting risk factors play a big role in the high frequency and severity of OSA in these patients and can explain the heterogeneity among different patient populations. Well-designed prospective and long-term studies should help us examine well the effect of the different risk factors of OSA in DS and evaluate the effectiveness of different diagnostic and treatment options.
